# A Criticism with Clinical Notes

**Published:** 1891-02

**Authors:** F. L. Griffith

**Affiliations:** Edina, Mo.


### A CRITICISM WITH CLINICAL NOTES.

F. L. GRIFFITH, M. D., EDINA, MO.

I want to ask a question or two of one or two contributors to the December number of this journal.

In the article headed, " Nuggets," by Dr. C. Carleton Smith, I notice many fine pointers, and at least one indication that does not jibe with my idea of things. In the 6th line from bottom of page 551, he says: " Phos. is worse lying on right side." Now I have relieved and cured many bad conditions of chest with Phos., and have always found it impossible for patient to lie on left side.

In Lippe's *Repertory* I find Phos. in italics as a remedy worse from lying on left side.

I will also have to ask an explanation of my Missouri colleague, Dr. Steinrauf, of St. Charles. He tells of a clinical case which he treated during the grippe epidemic last *March*, but he winds up the article by saying this cure was *two years ago*.

This is found in December number, page 557. If our friend does not correct this some of us might accuse him of inconsistency.

I am glad to notify the profession that my preceptor, Dr. H. S. Strickland, of Kirksville, Mo., has been appointed on the Board of Pension Examiners for this district. This doctor is no mongrel, but as pure a follower of the law as we have in the State.

I like to see the pure homœopath get to the front.

Now I must give a case or two from my own practice.

Three months ago I received a dispatch to come on first train to a town sixteen miles east of here. I was met at the depot by a very anxious husband and escorted to the scene of suffering. When I entered the room, Bry. entered my mind.

Here is the history :

Two weeks previously, caught cold and had quite a tormenting cough, stiff neck, worse right side. (You are not thinking of Bry., are you?) Well, the allopath was called and gave Quinine. About a week previous to my being called she was extremely bad with acute darting pains in right chest, every breath aggravated and a full breath would make her scream. For six days and nights she suffered intense agony, during which time she neither ate nor slept at all. The old doctor was there almost continually and Calomel, Quinine, and Morphine were the implements with which he was killing her. During all this time she could not move on account of the fearful aggravation. She lay continually on the painful side and drank enormous quantities of cold water. The old doctor called it pleuropneumonia and said she was very dangerously sick.

Would any homœopath call so plain a case dangerous? Any homœopathic student could have cured that case. She just had time to take three doses of Bry.$^{30}$ before going to sleep. She slept well all night and was entirely well in twenty-four hours. She only got three doses, thirty minutes apart. Now this happened three months ago, and I have made over two hundred dollars in cash out of that town since.

CASE II.—Was called eight miles to see a lady with violent chills.

She had the tertian type, great emaciation and prostration, yet she had good appetite. Violent headache and thirst before chill. Said she could not live through another such chill. Chill began in fingers and toes, blue lips and nails, nausea and horrible headache. Unconscious part of time.

Fever blisters full of clear, transparent fluid. Well I poured a few pellets from my Nat-m.$^{5000}$ bottle and left no medicine nor blanks. She seemed much surprised when I told her I would leave her no medicine, but she never chilled nor had any symptoms of chill after that one dose of weak salt. I just want to say a word here for the benefit of all those young homœopaths like myself who find it very hard to rely on one dose of the properly selected drug in a high potency.

I have been honestly testing the matter for the past three years, and although I find it hard to keep from repeating, I *know* I get better results if I do not.

I was called, not long since, to see a young lady afflicted with a disease called allopathy. In the first place, she had a fever for which the allopath gave three or four large doses of Calomel. The fever stopped but instead she got fearful pains in upper extremities, the pains being very much worse at night. At times the saliva would run out of mouth in a stream. Very fetid breath and large, flabby, yellowish tongue. She had been suffering so for five days and nights when I was called. The tongue said Merc., the breath said Merc., the saliva said Merc., and the great aggravation at night said Merc.

So she got Merc-sol.$^{6000}$, three doses twenty minutes apart, and plenty of Sac-lac. to follow. She began feeling better in less than two hours and in four days was entirely well. Now, my friends, in coming right down to facts, all we have to do is to be sure we get *all* the symptoms exactly, then find the simillimum, to do which requires *study, study!* Well, then, don't neglect to study.

